# A New Mouse Allele of Glutamate Receptor Delta 2 with Cerebellar Atrophy and Progressive Ataxia

**DOI:** 10.1371/journal.pone.0107867

**Published:** 2014-09-24

**Authors:** Yuka Miyoshi, Yoshichika Yoshioka, Kinuko Suzuki, Taisuke Miyazaki, Minako Koura, Kazumasa Saigoh, Naoko Kajimura, Yoko Monobe, Susumu Kusunoki, Junichiro Matsuda, Masahiko Watanabe, Naoto Hayasaka

**Affiliations:** 1 Department of Anatomy and Neurobiology, Kinki University School of Medicine, Osaka-Sayama, Osaka, Japan; 2 Biofunctional Imaging Laboratory, Immunology Frontier Research Center, Osaka University, Suita, Osaka, Japan; 3 Neuropathology, Tokyo Metropolitan Institute of Gerontology, Tokyo, Japan; 4 Department of Pathology and Laboratory Medicine, School of Medicine, University of North Carolina at Chapel Hill, Chapel Hill, North Carolina, United States of America; 5 Department of Anatomy, Hokkaido University Graduate School of Medicine, Sapporo, Japan; 6 Laboratory of Experimental Animal Models, National Institute of Biomedical Innovation, Ibaraki, Osaka, Japan; 7 Department of Neurology, Kinki University School of Medicine, Osaka-Sayama, Osaka, Japan; 8 Research Center for Ultra-High Voltage Electron Microscopy, Osaka University, Ibaraki, Osaka, Japan; 9 Section of Laboratory Equipment, National Institute of Biomedical Innovation, Ibaraki, Osaka, Japan; 10 Precursory Research for Embryonic Science and Technology (PRESTO), Japan Science and Technology Agency (JST), Kawaguchi, Saitama, Japan; Georgia Regents University, United States of America

## Abstract

Spinocerebellar degenerations (SCDs) are a large class of sporadic or hereditary neurodegenerative disorders characterized by progressive motion defects and degenerative changes in the cerebellum and other parts of the CNS. Here we report the identification and establishment from a C57BL/6J mouse colony of a novel mouse line developing spontaneous progressive ataxia, which we refer to as *ts3*. Frequency of the phenotypic expression was consistent with an autosomal recessive Mendelian trait of inheritance, suggesting that a single gene mutation is responsible for the ataxic phenotype of this line. The onset of ataxia was observed at about three weeks of age, which slowly progressed until the hind limbs became entirely paralyzed in many cases. Micro-MRI study revealed significant cerebellar atrophy in all the ataxic mice, although individual variations were observed. Detailed histological analyses demonstrated significant atrophy of the anterior folia with reduced granule cells (GC) and abnormal morphology of cerebellar Purkinje cells (PC). Study by ultra-high voltage electron microscopy (UHVEM) further indicated aberrant morphology of PC dendrites and their spines, suggesting both morphological and functional abnormalities of the PC in the mutants. Immunohistochemical studies also revealed defects in parallel fiber (PF)–PC synapse formation and abnormal distal extension of climbing fibers (CF). Based on the phenotypic similarities of the *ts3* mutant with other known ataxic mutants, we performed immunohistological analyses and found that expression levels of two genes and their products, glutamate receptor delta2 (*grid2*) and its ligand, cerebellin1 (*Cbln1*), are significantly reduced or undetectable. Finally, we sequenced the candidate genes and detected a large deletion in the coding region of the *grid2* gene. Our present study suggests that *ts3* is a new allele of the *grid2* gene, which causes similar but different phenotypes as compared to other *grid2* mutants.

## Introduction

Ataxia is defined as a neurological dysfunction that causes loss of motor coordination such as gait imbalance associated with appendicular ataxia and defects in gaze or speech [1]–[3]. Inherited spinocerebellar degenerations (SCDs) are among the main causes of ataxia. SCDs comprise the two most relevant forms of ataxia: the autosomal recessive ataxias and the autosomal dominant spinocerebellar ataxias (SCAs) [1]. The majority of recessive ataxias are caused by loss-of-function (missense) mutations, while SCAs are mostly caused by an insertion of multiple CAG-repeats in the coding region of a particular gene, which is thought to result in a toxic gain-of-function of the protein with poly-glutamine (poly-Q) expansion [1]. Another group of inherited SCDs is the hereditary spastic paraplegias (HSPs, numbered as SPG1-39) characterized by progressive lower limb spasticity and weakness due to distal axonopathy of the corticospinal tract axons [4], [5]. Over the last two decades, genetic studies have identified many genes responsible for the inherited SCDs, including 19 genes out of 27 known SCAs and 20 out of 36 established HSPs [3], [5].

Increasing numbers of the animal SCD models representing both sporadic mutant mice and genetically-engineered mice have been reported [6]–[8], including *hotfoot* and *Lurcher* mutants [9], [10]. Their causative mutations were identified in the same gene, *grid2*, encoding the ionotropic glutamate receptor delta-2 (GluD2) [11], [12], which is selectively expressed in Purkinje cells (PC). At least twenty alleles of the same gene, including *ho4J, ho5J, ho7J, ho8J, ho9J, ho11J, ho12J, ho13J, ho15J, tpr (tapdancer)*, 153Gso and 154Gso have been identified from different mutant mice [11], [13]–[16], and a targeted null mutation in the *grid2* gene (*grid2* KO mice) has also been reported [17]. These *grid2* mutants are commonly characterized by cerebellar atrophy and motor incoordination.

*Lurcher (Lc)* is a spontaneous semi-dominant mutation in which homozygotes (*Lc/Lc*) die at birth, and heterozygotes exhibit cerebellar deficiencies and ataxia [9], [10]. It is the result of a single missense mutation in the third transmembrane domain in the *grid2* gene [11]. *Lc* is an autosomal dominant and constitutive active mutation which eventually leads to the death of PCs, resulting in their near complete absence as well as the accompanying loss of most GCs and 60–75% of olivary neurons [12], [18].

*Hotfoot* (*ho*) is a spontaneous, autosomal recessive mutation, and one of the alleles, *ho4j*, was first shown to be caused by a mutation in the *grid2* gene [11]. In addition to the ataxic phenotypes common to *Lc/*+, both *hotfoot* mutants and *grid2* KO mice are characterized by deficits in parallel fiber (PF)-PCs and climbing fiber (CF)- PC synapse formation, as well as impaired induction of long-term depression (LTD) [16], [17]. Phenotypic similarities between *hotfoot* mutants and *grid2* KO mice suggest that *hotfoot* is a loss-of-function mutation. In fact, sensorimotor learning deficits exhibited by *hotfoot* mutants are greater than those demonstrated by *Lc/*+ mutants[19], [20], although loss of cerebellar cells in *hotfoot* mutants is less severe [18], [21].

Here, we have established a mouse line with an autosomal recessive gene mutation characterized by progressive ataxia and significant cerebellar atrophy. Phenotypic and genetic analyses suggest that the mutation is a new allele of the *grid2* gene and another loss-of function mutation.

## Materials and Methods

### Mice

Experimental protocols for mice were approved by committees at the National Institute of Biomedical Innovation (DS 23-35), Kinki University (KAME-22-012), Osaka University (FBS 07-001). Mice were maintained under standard conditions of light (8:00 am–8:00 pm) and temperature (23+/−1°C). The *ts3* mutant was originally found in the C57BL/6 strain, and additional C57BL/6J mice used for all experiments, including mating, were obtained from SLC Japan. All surgery was performed under 1% isoflurane anesthesia or pentobarbital anesthesia (100 mg/kg body weight, i.p.), and all efforts were made to minimize suffering.

For *in vitro* fertilization, 4-week-old female C57BL/6J mice were intraperitoneally injected with 5 IU pregnant mare serum gonadotropin (PMSG; Serotropin, ASKA Pharmaceutical), followed by 5 IU human chorionic gonadotropin (hCG; Puberogen, Yell Pharmaceutical) 48 h later. Fifteen hours after hCG injection, oocytes were dissected from the ampulla region of the oviducts and placed in 200 µl droplets of HTF medium (Ark Resource) at 37°C under 5% CO_2_ in air. Spermatozoa collected from the cauda epididymis of *ts3* males were incubated for 1–1.5 h in 200 µl droplets of HTF medium to allow capacitation. Oocytes were then inseminated by adding 3–5 µl of the sperm suspension and incubated for 5 h. The fertilized oocytes were washed three times and transferred to fresh drops of KSOM medium (Ark Resource) and cultured overnight. The following day, the 2-cell stage embryos (usually 10 embryos/oviduct) were surgically transferred into the oviducts of pseudopregnant ICR females (0.5 day post coitus) that had been mated with vasectomized males.

When taking footprints, soles of the hind feet were marked with Chinese ink, and the mice were allowed to walk freely on flat paper.

### Micro-magnetic resonance imaging (micro-MRI)

An MRI apparatus for small animals at 11.7 T (Bruker, AVANCE 500WB) was used for the experiments. Mice (n = 6) were anesthetized with 1% isoflurane and MRI was performed under different sequences as follows:

1. Sequence for brain imaging: T_1_ weighted image (Gradient echo: FLASH);

FOV = 2.0 cm, matrix  = 256×256, thickness  = 0.5 mm, 12 slices,

TR/TE  = 100 ms/2.3 ms, NS = 32, scan time  = 13 min. MRI was performed 20∼24 h after IP injection of Mn solution (0.4 mmol/kg MnCl_2_/4H_2_O, 100 mM).

2. Sequence for spinal cord/sciatic nerve imaging:

2-1) T_2_ weighted image (Spin echo: RARE)

FOV  = 2.5 cm, matrix  = 256×256, thickness  = 0.5 mm, 12 slices,

TR/TE  = 4000 ms/25.2 ms, NS  = 8, scan time  = 8 min.

2-2) Diffusion tensor image (Spin echo)

FOV  = 2.5 or 3.0 cm, matrix  = 128×128, thickness  = 0.5 mm, 8 slices,

TR/TE  = 2000 ms/27 ms, b  = 1000 s/mm^2^, 6 directions, NS = 4, scan time  = 119 min.

### Histology

Mice at the ages of 3 weeks, 4 months and 10 months were used for histological studies. Following the MRI scan, mice were anesthetized with pentobarbital and perfused with 4% paraformaldehyde. The brain was then removed, embedded in paraffin, and used for histological analysis. The sections were stained with hematoxylin-eosin (H&E), toluidine blue, Kluver-Barrera's (KB) stain, following standard protocols.

### Immunohistochemistry

Under pentobarbital anesthesia, mice were fixed transcardially with 4% paraformaldehyde in 0.1 M sodium phosphate buffer (PB, pH 7.2), and cerebellar sections (50 µm in thickness) were prepared using a microslicer (VT1000S, Leica). Immunohistochemical incubations were done at room temperature using phosphate-buffered saline (PBS, pH 7.2) containing 0.1% TritonX-100 (TPBS) for diluent and washing buffers. For immunofluorescence, most sections were successively incubated in a free-floating state with 10% normal donkey serum for 30 min, a mixture of primary antibodies overnight (1 µg/ml), and a mixture of Alexa Fluor-488-, indocarbocyanine (Cy3)-, and indodicarbocyanine (Cy5)-labeled species-specific secondary antibodies (1∶200, Invitrogen; Jackson ImmunoResearch) for 2 h. For immunohistochemical analysis with calbindin antibody, we used fresh frozen sections mounted on silane-coated slide glasses which were fixed with 4% PFA for 10 min and then subjected to immunofluorescence incubation as above. Images were taken with a laser-scanning microscope (FV1000, Olympus) equipped with HeNe/Ar laser, and PlanApo (10x/0.40) and PlanApoN (60x/1.42, oil immersion) objective lens (Olympus). To avoid cross-talk between multiple fluorophores, Alexa 488 (or FITC), Cy3, and Cy5 fluorescent signals were acquired sequentially using the 488 nm, 543 nm, and 633 nm excitation laser lines. Single optical sections were obtained (640×640 pixels, pixel size 110 nm).

For immunohistochemical analysis, we used affinity-purified primary antibodies raised against the following molecules (host species): mouse calbindin (goat, [22]), mouse cerebellin 1 (cbln1, guinea pig, [23]), mouse glial fibrillary acidic protein (GFAP, rabbit, [24]), mouse parvalbumin (guinea pig, [25]), mouse glutamate receptor GluD2 (rabbit, [26]), rat vesicular glutamate transporters VGluT1 and VGluT2, (guinea pig, [27]), and mouse vesicular inhibitory amino acid transporter (VIAAT, guinea pig, [27]).

### Electron microscopy

Brains from 10-month-old (30 weeks) mice were perfused with 4% paraformaldehyde/2.5% glutalaldehyde and fixed for 2 hours in the fixative solution. The cerebella were cut in small pieces (about 1 mm^3^) and washed with phosphate buffer, postfixed in sodium cacodylate-buffered 1.5% osmium tetroxide for 60 min at 4°C, dehydrated using a series of ethanol concentrations, and embedded in Epon resin. The samples were examined under an electron microscope (H-7650, Hitachi or JEM1400, JEOL).

### Ultra-high-voltage electron microscopy (UHVEM)

An ultra-high voltage (3MV) electron microscope (H-3000, Hitachi) was used. Mouse brain (4 months old) was fixed with 4% paraformaldehyde/2.5% glutalaldehyde solution overnight and sagittal brain slices (500 µm thickness) were prepared using Microslicer (Matsunami). Golgi stain was performed on the slices following standard protocol. Following several rinses in 8% sucrose in phosphate buffered saline (PBS), the slices were immersed for 2 h in a mixture of 2.5% paraformaldehyde, 2.5% glutalaldehyde in PBS. After fixation with 1% osmium tetraoxide for 1 h, the thick sections were dehydrated through a graded series of ethanol (60%–100%) and propylene oxide. Finally, they were embedded in Quetol 812 at 45°C for 1 day, and then at 60°C for 2 days. The embedded sections were mounted on epoxy block and cut on an ultramicrotome (Ultracut E; Reichert-Jung) at 10 µm thickness and mounted on 50-mesh grids. Specimens were examined with an ultra-high voltage electron microscope H-3000 (Hitachi) at an accelerating voltage of 2000 kV. Images were recorded with a 4 k×4 k slow scan charge-coupled device (SSCCD) camera F486BK with a pixel size of 15 µm×15 µm (Hitachi) at a nominal magnification of 2,000×.

### RT-PCR

Reverse transcription PCR (RT-PCR) was performed according to standard protocol. Briefly, cerebella were sampled from *ts3* mice and wild-type controls. Total RNAs were extracted using Trizol reagent (Thermo Scientific) and cDNAs were synthesized from the RNAs using Superscript II Reverse Transcriptase (Life Technologies) with either oligo (dT) or random primers (Life Technologies), according to the manufacturers’ protocols. Nested PCR was performed to amplify the *grid2* cDNA from *ts3* and wild-type cerebella using Ex-Taq (TaKaRa) and primers as follows: 5’-ATGGAAGTTTTCCCCTTGCT-3’, 5’-TCATATGGACGTGCCTCGGTCG -3’. PCR parameters are as follows: 94°C 2 min; 30 cycles of 94°C 1 min, 60°C 1 min; 72°C 3 min, 72°C 7 min.

### Western blotting

Western blotting was performed according to standard protocol. Briefly, sampled cerebella were homogenized in RIPA buffer with protein inhibitors and proteins were extracted by sonication and centrifugation. 10 µg of each protein was electrophoresed using SDS-PAGE gel, blotted on PVDF membrane (Bio-Rad), and incubated overnight at 4°C with anti-GluD2 antibody (1: 2000 dilution, rabbit, [26]), anti-Cbln1 antibody (1: 250 dilution, rabbit, [23]), anti-α-tubulin antibody (1: 10,000 dilution, rabbit, MBL) or anti-β-actin antibody (1: 400,000 dilution, mouse, Sigma). Secondary antibodies, anti-rabbit IgG (donkey, GE Healthcare) or anti-mouse IgG (sheep, GE Healthcare) were used for detection of anti-rabbit (GluD2, Cbln1, or α-tubulin) or anti-mouse (β-actin) primary antibody, respectively. ECL Prime Western blotting reagent (GE Healthcare) was used for chemiluminescence detection and images were captured using MYECL Imager (Thermo Scientific).

### Statistics

All data are expressed as the mean ± SEM (or SD if specifically described). Statistical significance was evaluated by Student’s *t*-test and was set at the *p*<0.05 level, except in a case where chi-square (χ^2^) analysis was performed to compare frequency distribution of dendritic spine morphology between WT and *ts3* mice in the four different categories.

## Results

### Isolation of a mouse line with behavioral abnormality

**Figure 1:**
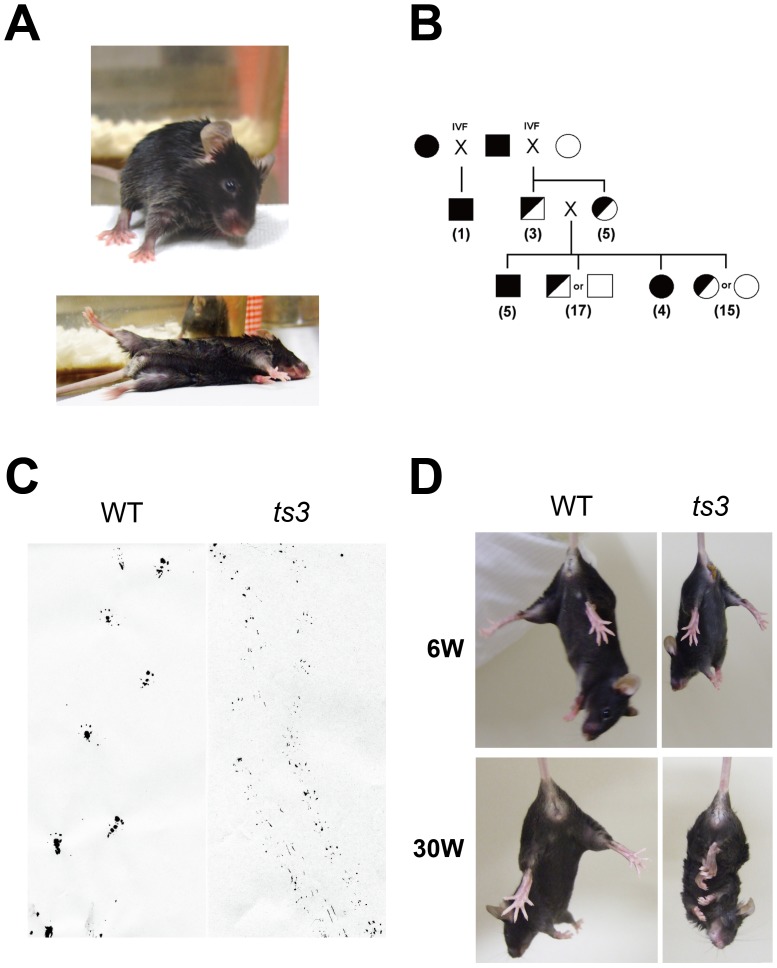
Isolation of a mouse line with behavioral abnormalities. **A)** Mutant mice fall frequently and display an inability to right themselves easily. **B)** Determination of hereditary pattern by IVF. Squares and circles indicate male and female, respectively. Black-fill indicates mice, possibly homozygote of the mutated allele, with behavior abnormalities. Half-filled and open symbols are presumed to be heterozygote and WT with normal behavior. Numbers indicate number of offsprings. **C)** Hind heel footprints for normal and mutant mice. **D)** Frequent limb-clasping displayed by older mice (30-week old).

While maintaining the C57BL/6J mouse population, we discovered some mice with the ataxic phenotype. The mice could not move smoothly and also fell frequently, most likely due to abnormal control of their hind limbs (Fig. 1A). To clarify hereditary mode of the ataxic phenotype, the mice were mated with normal C57BL/6J mice. However, both male and female failed to produce offspring under natural breeding conditions, presumably due to ataxia. Consequently, we performed in vitro fertilization (IVF) in order to obtain offspring.

As shown in Fig. 1B, one male mouse with ataxia was born via IVF using sperm from a mutant male and oocytes from a mutant female. In contrast, when oocytes from normal females were used for IVF, no offspring displayed abnormality at F_1_ generation. At F_2_ generation, however, about 25% of offspring exhibited the same phenotype as the original mice, suggesting that the ataxic phenotype is recessively inherited and that a single gene is responsible for the phenotype. We referred to the mutant mice as *ts3*.

### Progressive changes in abnormal behavior

To characterize the behavioral abnormalities of the *ts3* mice more precisely, we first collected footprints of hind soles from mutants and control littermates (Fig. 1C). The mutant mice displayed a short-stepped gait and moved their toes parallel to the direction of movement. Moreover, the *ts3* mice did not attach their heels on the ground, suggesting that this behavioral abnormality is one of the characteristics of spinocerebellar ataxia. Although the extent of hind limb abnormality was not significant during suckling period (2 weeks old), after weaning (3 weeks old) the mutant mice began to fall frequently. Finally, at 6-weeks, the mice began to walk dragging their hind limbs.

Because mice with spinocerebellar ataxia display frequent limb-clasping, normal and mutant mice were examined by hanging test. When young mice (6-week old) were tested, both normal and *ts3* mice were well balanced by opening their limbs (Fig. 1D, upper panel). Some of the older *ts3* mice (i.e. after 12 weeks; the figure shows 30-week-old mice), however, exhibited frequent limb-clasping (Fig. 1D). In addition, tail-muscle tonus (not shown) and hind-limb tonus were observed in older *ts3* mice after having an object stuck between the thigh and the abdomen, suggesting that the mutant is a potential model for recessive spinocerebellar ataxia and that pathological abnormality progresses after weaning. Interestingly, the *ts3* mice showed characteristic traits of paraplegia but no sign of spasticity, suggesting that the mutants should not be considered a model for human hereditary spastic paraplegia (HSP). We also found that the ataxic *ts3* mice were significantly smaller than normal littermates (Fig. S1).

### Identification of cerebellar atrophy in *ts3* mice by Micro-MRI

**Figure 2:**
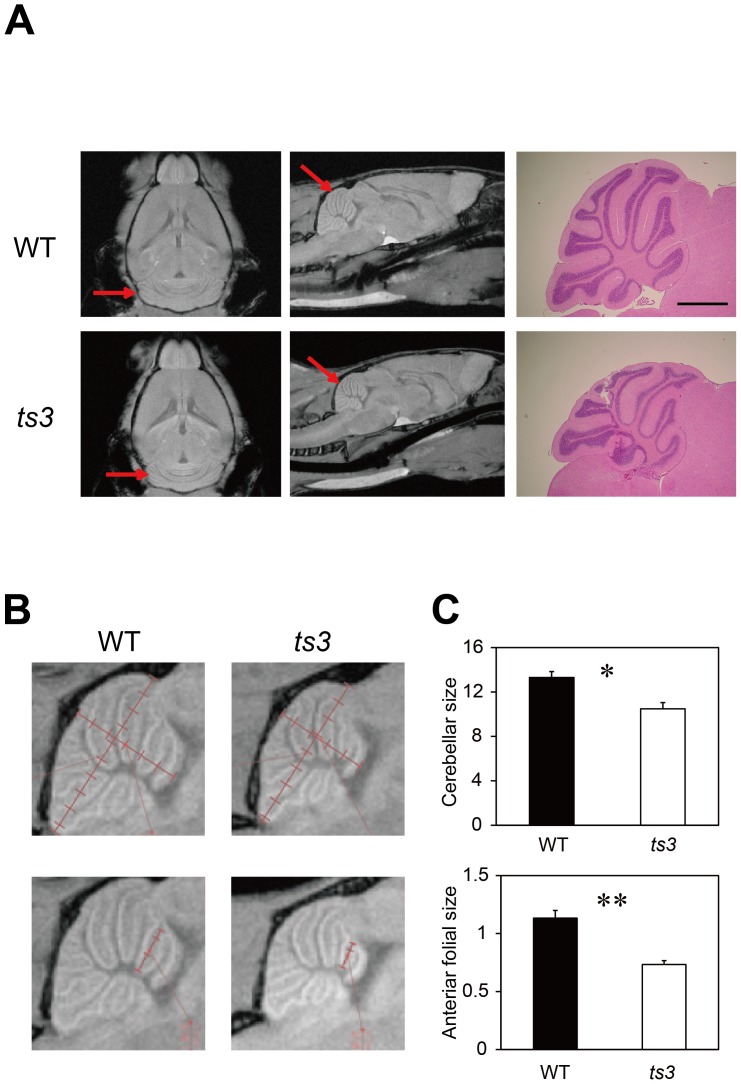
Cerebellar atrophy in the *ts3* mutant mice revealed by Micro-MRI and histological analysis. **A)** Brain images acquired by micro-MRI (left and middle panels). Horizontal (left) and sagittal (middle) images of cerebella indicate cerebellar atrophy in the *ts3* mutant (lower panels) and WT control (upper panels). The right panels show H&E-stained cerebellar sagittal sections of the *ts3* mutant (lower) and WT control (upper). Scale bar indicates 1 mm. **B, C)** Cerebellar size of *ts3* mutants and WT controls. First, the lengths of major and minor axes (mm) of the whole cerebellum were measured by micro-MRI (B, upper panels). They were consequently multiplied and compared between two genotypes (C, upper, n = 6, unit: mm^2^). To examine significant anterior folial atrophy in *ts3* cerebella, we also measured the length of the folia (shown in B, lower) and compared in *ts3* and WT mice (C, lower, n = 6, unit: mm). *P<0.05, **P<0.01.

**Figure 3:**
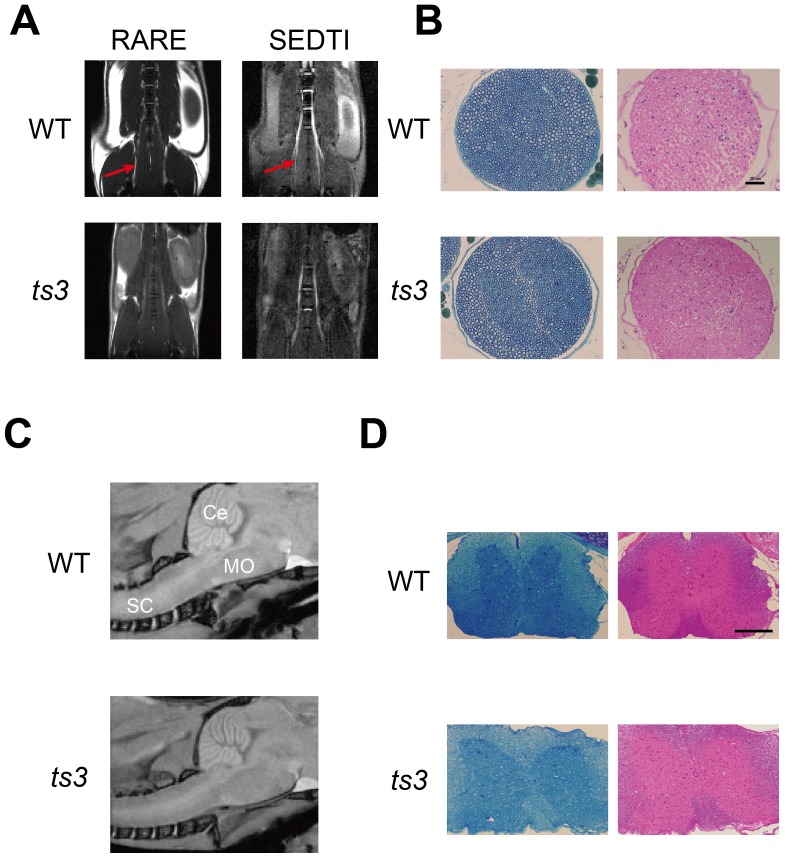
Normal morphology for spinal cord and sciatic nerves. **A)** No significant abnormality in the sciatic nerve (arrow) was detected in *ts3* mutants by micro-MRI. **B)** Toluidine blue (left) and H&E staining (right) shows normal morphology of sciatic nerves in both genotypes. Scale bar: 25 µm. **C)** Micro-MRI sagittal images of brain stem and spinal cord in *ts3* and WT mice. No abnormalities were detectable in *ts3*. Ce: cerebellum, MO: medulla oblongata, SC: spinal cord. **D)** Toluidine blue and H&E-stained spinal cord of *ts3* and WT mice. Spinal cord morphology was normal in both genotypes, and no significant difference was observed. Scale bar indicates 100 µm.

To investigate the cause of ataxia in the *ts3* mice, we first performed micro-MRI on *ts3* and WT brains. We found that the cerebella of *ts3* mutant mice were significantly smaller than that of WT controls (4 months old, n = 6 each, Fig. 2A, B), measuring the size of different regions of the cerebella for both genotypes and confirming significant cerebellar atrophy in the *ts3* mutants (Fig. 2C, D, Fig. S2). We also performed histological analyses on the same animals, with results consistent with those from micro-MRI (Fig. 2A, B). Finally, we examined micro-MRI and histology of the spinal cords and sciatic nerves of the *ts3* mice when compared to the WT controls (4 months old, n = 3 each, Fig. 3, Fig. S3). No detectable abnormality (e.g., demyelination or degeneration of upper/lower neurons) was observed in either sciatic nerves (Fig. 3A, B) or spinal cords (Fig. 3C, D, S3) of the *ts3* mutants.

### Abnormality in the Purkinje and granule cells of the anterior folia in *ts3* cerebella

**Figure 4:**
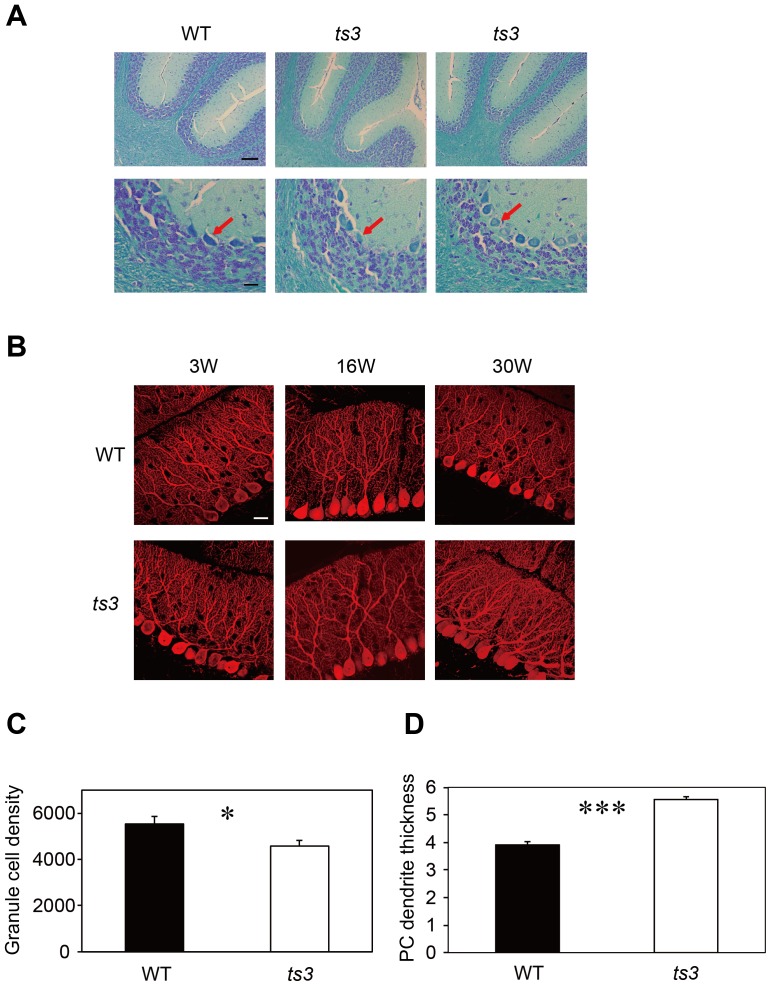
Morphological abnormality of Purkinje cells in *ts3* cerebella. **A)** Kluver-Barrera's stain was performed on *ts3* and WT cerebella. Number and morphology of Purkinje cells were normal in *ts3* mutants. In contrast, however, GC numbers were reduced, and the size of the nuclei was significant smaller than WT. Scale bars, 50 µm (upper panel); 20 µm (lower panel). **B)** Immunohistochemisty was performed on cerebella in *ts3* and WT mice using anti-calbindin antibody as a Purkinje cell marker. Purkinje cell morphology became progressively altered commensurate with mouse age. Note that dendrites of *ts3* Purkinje cells at 30 weeks of age look significantly thicker than their WT counterparts. Dendrite branching was also abnormal in *ts3* mutants. Scale bar, 20 µm. **C)** Significant reduction in granule cell density (cell number/mm^3^) in old *ts3* mutants (30 weeks old). **D)** The PC dendrite is significantly thicker in *ts3* mutants at 30 weeks of age (n = 70 each, averaged diameters of the thickest regions). *P<0.05, ***P<0.001.

The atrophy observed in the *ts3* cerebella was further examined histologically. Fig. 4 shows KB-stained sections of the *ts3* and WT cerebella. Reduced numbers of GCs were observed in older *ts3* mutants when compared to the WTs (30 weeks old, Fig. 4A, C). Next, we performed immunohistochemistry using anti-Calbindin antibody to examine PC morphology. As shown in Fig. 4B, dendrite branching of PCs in the molecular layer of *ts3* cerebella was abnormal, and each dendrite was significantly thicker in the 30-week-old mutants than in WT control (Fig. 4B, D). Average number of PCs in each lobule was also calculated from serial sections, and significant decreases were observed in lobules VII and VIII in the 30-week-old *ts3* mutants (Fig. S4).

### Electron microscopic analysis of Purkinje cells in *ts3* mutants

**Figure 5:**
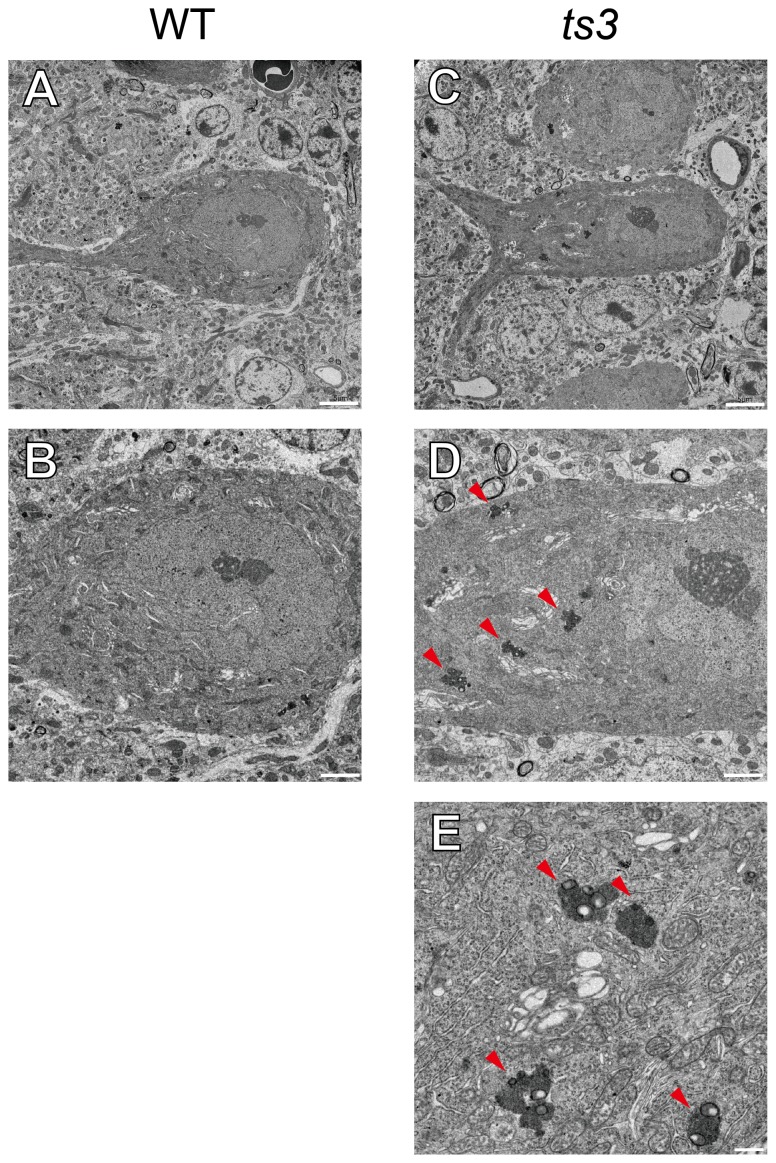
*ts3* purkinje cells demonstrate a characteristic of senescent cell as observed by electronmicroscopy. Electron photomicrographs of PC cells in 30-week-old WT (**A**, **B**) and *ts3* (**C**, **D**, **E**). Lipofuscin accumulation in cell bodies (arrowheads in **B** and **C**), representing senescent postmitotic neurons, was observed in *ts3*, but not in WT PC cells. Scale bars, A, B, 5 µm; C, D, 2 µm; E, 0.5 µm.

To further analyze PC abnormality in the *ts3* cerebellum, we performed an electron microscopic study on mutant and normal cerebella. In *ts3* cerebella, we observed electron dense accumulations in cell bodies of PC cells (30 weeks old, Fig. 5B, D, E, arrowheads). Higher magnificent images demonstrated that the aggregates contain electron dense and electron lucent compartments (Fig. 5E), which are characteristics of lipofuscin. Lipofuscin is composed of highly oxidized cross-linked protein aggregates and lipids. Previous reports suggest that lipofuscin accumulation is a consequence of oxidative stress and is a characteristic feature of senescent postmitotic cells including neurons [28]. These granules were not observed in WT PC cells (Fig. 5A, B).

### Abnormal morphology of dendrites and spines in *ts3* mutants revealed by ultra-high voltage electron microscopy

**Figure 6:**
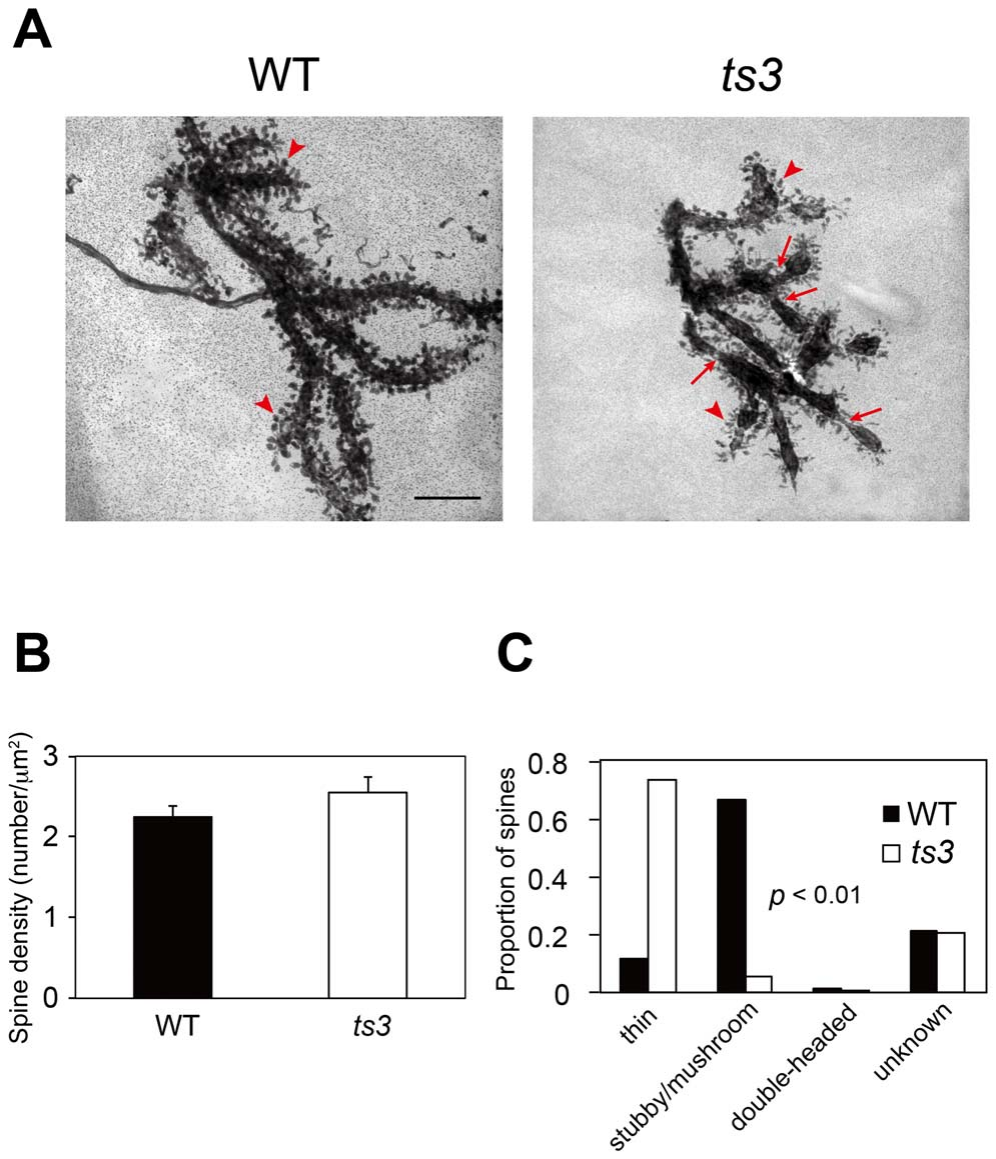
Abnormal morphology of dendrites and spines of the Purkinje cells in *ts3* mutants. **A)** Representative ultra-high voltage electron microscopy (UHVEM) images of Purkinje cell dendrites in *ts3* mutant and WT mice (2000x). The arrow in the lower panel indicates isthmic portions of dendrites observed in *ts3* mutants. Note the significant difference in morphology between *ts3* and WT cerebella. Whereas the bulbous shape of dendritic spines is observed in WT controls, dendritic spines in *ts3* mutants are smaller and irregularly-shaped, as indicated by arrowheads. Scale bar indicates 5 µm. **B)** Dendritic spine density (number of spine/µm^2^) was comparable in *ts3* and WT controls. **C)** Morphology of the dendritic spines is significantly different in *ts3* mutants when compared to that of WT controls. Whereas stubby or mushroom-shaped spines, which are characteristic of normal and mature PCs, are commonly observed in WT cerebella, thin (filopodia-like or headed) spines, representing a deficit in spine maturation, are predominant in *ts3* mutants. Double-headed spines, including branched spines, were rarely observed in either genotype. “Unknown” indicates spines that could not be categorized from HVEM images by gross observation due to high density of dendritic spines in PCs. The χ^2^ test indicated that the difference in dendritic morphology between two genotypes is statistically significant (*p*<0.01).

We next performed ultra-high voltage electron microscopy to further analyze abnormalities in PCs of the *ts3* mutants. As shown in Fig. 6A (indicated by arrowheads), morphology of the dendrites and dendritic spines in the PCs of the *ts3* mutants differed from those in the WT cerebella (4 months old). To quantify the difference in dendritic spine morphology, we compared spine density (Fig. 6B) and shapes (Fig. 6C) according to previous report [29]. Dendritic spine density was comparable between *ts3* and WT controls (Fig. 6B), however, frequencies of spine morphology, which was categorized as thin, stubby, mushroom and double-headed, were significantly different between the two genotypes (Fig. 6C, *P*<0.01). Strikingly, not only spines but also dendrites were malformed; unlike WT, the thickness of dendrites was not constant with isthmic portions at intervals observed in the *ts3* mutants (Fig. 6A, arrows). These morphological abnormalities have not been described in previous studies.

### Distal extension of climbing fiber territory in *ts3* mutants

**Figure 7:**
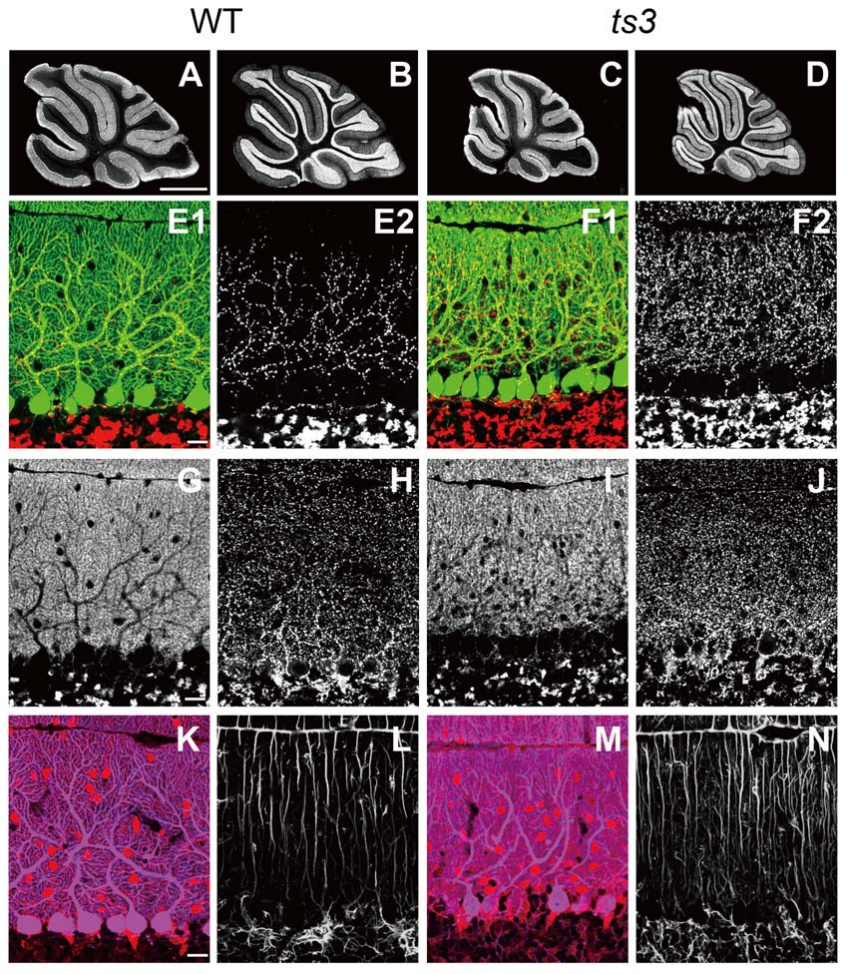
Distal expansion of CF territory in *ts3* mutants. **(A-N)** Immunofluorescence for calbindin (A, C, green in E, F, blue in K, M), VGluT2 (B, D, red in E, F), VGluT1 (G, I), VIAAT (H, J), parvalbumin (red in K, M) and GFAP (L, N) in control (A, B, E, G, H, K, L) and the *ts3* mutants (C, D, F, I, J, M, N). Note that VGluT2-positive CF terminals are markedly increased in number and distributed throughout the molecular layer in *ts3* mutants (E, F). Scale bars, A, 1 mm; E, G, K, 20 µm.

To investigate changes in cellular morphology and neuronal projection in the cerebellum of *ts3* mutants, we performed immunohistochemical studies using antibodies to calbindin, VGluT2 (vesicular glutamate transporter 2), VGluT1, VIAAT (vesicular inhibitory amino acid transporter), parvalbumin and GFAP (glial fibrillary acidic protein) (4 months old, Fig. 7). Through calbindin immunofluorescence, cerebella in *ts3* mutants was normal in foliation, laminated organization, and monolayer alignment of PCs, although cerebellar size was smaller than control mice as described in Fig. 2 (Fig. 7A-D). However, a striking phenotype was found in the distribution pattern of VGluT2-stained CF terminals. In control mice, CF terminals were distributed along calbindin-stained PC dendrites in the basal four-fifths of the molecular layer (Fig. 7B, E). In contrast, in the *ts3* mutants CF terminals were markedly increased in number and distributed throughout the full extent of the molecular layer (Fig. 7D, F). No such genotypic differences were noted for VGluT2-stained PF terminals (Fig. 7G, I), VIAAT-stained interneuron terminals (Fig. 7H, J), parvalbumin-stained inhibitory neurons (Fig. 7K, M) or GFAP-stained Bergmann fibers (Fig. 7L, N).

### Impaired PF–PC synapse formation in *ts3* cerebella

**Figure 8:**
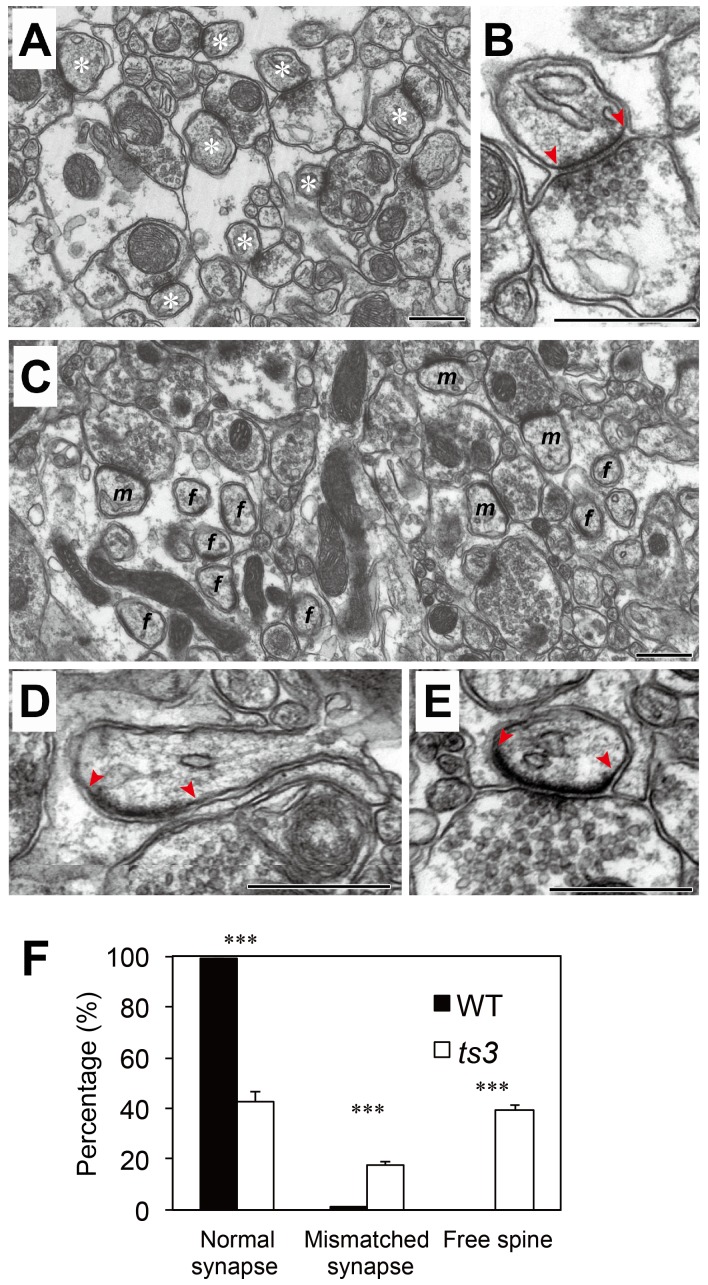
Free spines and mismatched PF-PC synapses in *ts3* mutant cerebella. **(A-E)** Conventional electron microscopy in control (A, B) and *ts3* mutant (C-E). **A)** Asterisks indicate PC spines contacting with PF terminals. **B)** Higher magnification image of a normal spine in WT. **C)** Letters *f* and *m* indicate free spines and mismatched PF–PC synapses, respectively. High-power images of free spine (D) and mismatched synapse (E). Note that PSD, as indicated between two arrowheads, is well-matched with the synaptic terminal in normal control (B). In contrast, the free spine and mismatched synapse do not have any contact with PF synaptic terminal (D) or has a significantly longer PSD (E), respectively. Scale bars, 500 nm. **F)** Percentage of normal synapses, mismatched synapses, and free spines in *ts3* and WT cerebella. Majority of spines observed in *ts3* cerebella were either mismatched or free spines. ***P<0.001.

Similar expanded distribution of CF terminals has been reported in mutant mice expressing defective glutamate receptor GluD2, Cbln1, or carbonic anhydrase-related protein 8 (Car8) [17], [30], [31]. In these mutants, PF-PC synapse formation is commonly impaired, as reflected in the emergence of ”free spines” lacking synaptic contact with PF terminals and of ”mismatched synapses”, in which the postsynaptic density (PSD) in PC spines is longer than the active zone in PF terminals [17], [31]–[33]. Therefore, we investigated the formation of PF–PC synapses in *ts3* mutants by electron microscopy (4 months old). In control mice, most PC spines contacted PF terminals in a one-to-one fashion, and the PSDs and active zones were well matched at PF-PC synapses. In *ts3* mutants, we frequently observed free spines (*f* in Fig. 8C, D) and mismatched synapses (*m* in Fig. 8C, E). Fig. 8F shows frequencies of normal synapses, mismatched synapses and free spines in the two genotypes. Whereas normal synapses were observed in about 99% of the PF–PC synapses in WT mice, the majority of the synapses/spines observed in *ts3* mutants were either mismatched (17.8±1.4%) or free (39.4±4.4%, *P*<0.001).

### Significant reduction of GluD2 and Cbln1 expression in *ts3* cerebella

**Figure 9:**
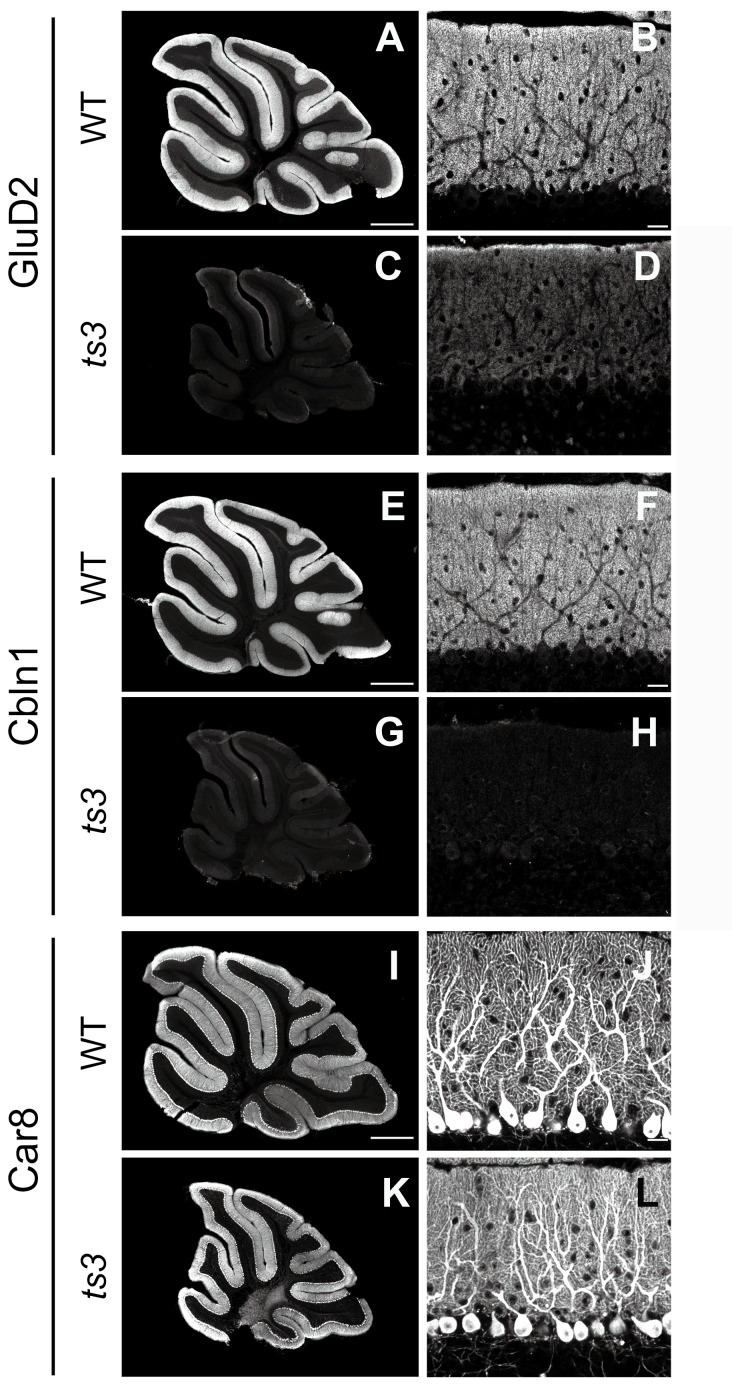
Severe reduction of GluD2 and Cbln1 immunoreactivities in *ts3* mutants. **(A-L)** Immunohistochemistry for GluD2 (A-D), Cbln1 (E-H) and Car8 (I-L) in control (A, B, E, F, I, J) and *ts3* mutants (C, D, G, H, K, L). Scale bars, A, E, I, 500 µm; B, F, J, 20 µm.

**Figure 10:**
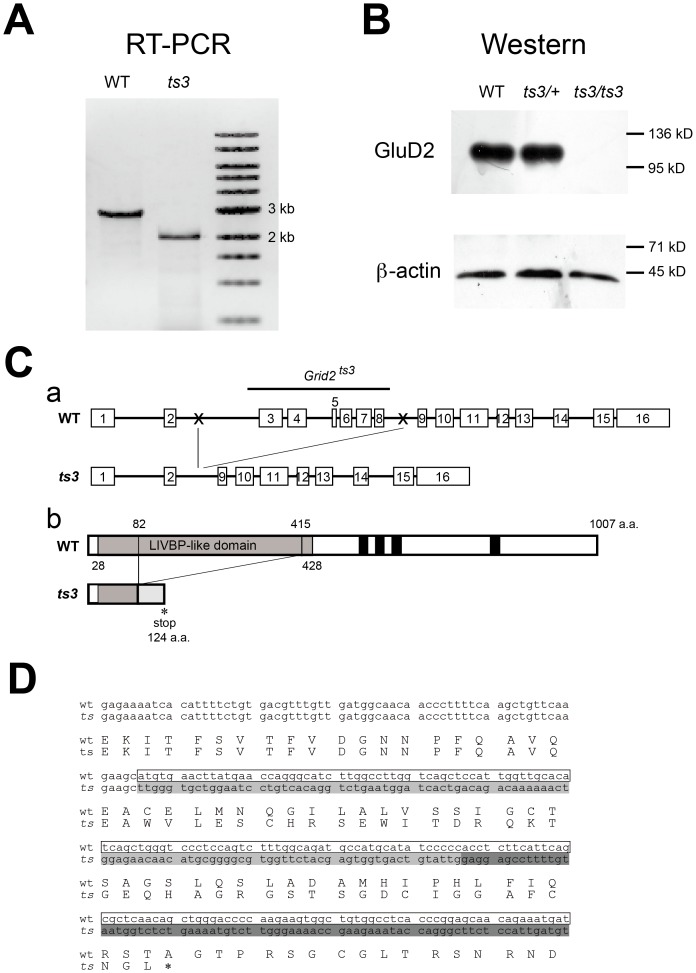
Identification of the *ts3* mutation in the *grid2* gene. **A**) RT-PCR was performed on mRNA extracted from either *ts3* or WT cerebella. A band that was roughly 1 kb shorter was detected from the mutant cDNA, suggesting a large deletion in the *grid2* DNA of *ts3* mice. **B**) Proteins were extracted from the *ts3* and WT cerebella and Western blotting was performed using anti-GluD2 and β-actin antibodies. The GluD2 protein was not detected in *ts3* mutant cerebella. **C**) Schematic representation of the deletion in *ts3* genomic DNA (a) and protein (b). **a**) The 6 exons (exon 3 to 8) are missing, and only portions of intron 2 and 9 were detected by genomic PCR (DNA sequences between the two Xs in the WT are missing in the *ts3* mutants). **b**) The deletion in the *grid2* DNA results in protein truncation (from amino acids 83 to 145) and also a frame-shift mutation. The resulting gene product in *ts3* mutants is 124 amino acids in length. **D**) Sequencing analyses confirmed that whereas exon 4 (enclosed sequences in the upper line) follows exon 3 sequences in the WT cDNA, exons 9 (shaded sequences in light gray) and 10 (shaded in dark gray) follow exon 3 in *grid2^ts3^*. The deletion in the *grid2^ts3^* gene causes a frame-shift mutation from amino acid 83 (C to W) and following amino acids in the GluD2^ts3^ protein and terminates the protein in a truncated, 124 amino acid form due to a termination codon (tga, *).

As the phenotypes in *ts3* mutants with abnormalities in PF–PC synapses are similar to those of GluD2, Cbln1 and Car8 mutants, we compared expression of these three gene products by immunohistochemistry (4 months old, Fig. 9). Severe reduction or loss of immunoreactivity was observed in GluD2 (Fig. 9A-D) and Cbln1 (Fig. 9E-H) but not Car8 (Fig. 9I-L), in the cerebella of *ts3* mutants. We also performed western blotting analyses and found that, whereas GluD2 expression was not detectable in *ts3* cerebella (Fig. 10B), expression level of Cbln1 was comparable in the mutant cerebella when compared with that of WTs (Fig. S5).

#### Identification of a large deletion of the *grid2* gene and protein in *ts3* mutants

Ultimately, we sequenced the two genes and also performed PCR on genomic DNA or cDNA from *ts3* and wild-type cerebella to investigate the possibility that a mutation in one of these genes might be responsible for the defects in *ts3* mutant mice. We detected a large deletion of exons in the *grid2* gene ranging from exon 3 to 8, resulting in a 1001 bp deletion in the *grid2* cDNA (Fig. 10A, C, D, S6A). By genomic PCR analysis, we also discovered the absence of the 3’ portion of the intron 2, suggesting the existence of a mutation within the intron 2 (Fig. 10C). Based on sequencing analyses, deletion of the *grid2^ts3^*DNA also resulted in a frame-shift mutation, causing a GluD2^ts3^ amino acid substitution from the amino acid 83 and following amino acids (Fig. 10C, D, S6B) and also termination of translation at the amino acid 124 (Fig. 10C, D). The results from Western blotting were consistent in that the GluD2^ts3^ protein was undetectable using an antibody that recognizes the C-terminus of GluD2^ts3^ (Fig. 10B).

## Discussion

The present study demonstrated that the aforementioned novel mutant mice exhibit progressive ataxia, with onset at approximately 3 weeks of age. The major symptoms of ataxia are paraplegia and gait imbalance, possibly due to hind limb incoordination. Cerebellar atrophy, morphological abnormality of PCs, reduced number of GCs and evidence for aberrant PF–PC synapse formation in *ts3* mutants strongly suggest that ataxia is caused by progressive dysfunction in the cerebellar network.

Morphological analyses in the present study revealed that PC dendritic spines were abnormal. Electron microscopic studies further demonstrated that PF–PC synapses were also abnormal in *ts3* mutants, with a large number of “mismatched synapses” and “free spines” being observed [34]. In normal PF-PC synapses, length of the postsynaptic density (PSD) of the PC spine is well-matched with that of the active zone of PF. In contrast, PSD in *ts3* mutants was significantly longer than the active zone of the PF terminal, which is similar to previous observations in *Car8*, *grid2*, and *Cbln1* mutant mice [30], [31], [33].

In previous studies, all three mutants demonstrated cerebellar dysfunctions, resulting in abnormalities such as motor dyscoordination, defects in PF–PC synapse formation, and expansion of CF territory. There are, however, differences in the phenotypes of these mutants when compared to that of *ts3*. For instance, *Car8* KO mice demonstrated expansion of CF territory and free spines, but mismatched synapses were not observed [30]. Phenotypes of *Cbln1*- and *grid2*-disrupted (KO) mice were similar according to previous reports, demonstrating motor dyscoordination, increase in the number and distribution of CF terminals, and significant rates of free/mismatched synapses [17], [31], [35]. However, our study indicated that there are also discrepancies between *ts3* mutants and *Cbln1-/grid2* KO mice, such as lower body weight, failure to produce offspring, and significant individual differences observed in *ts3* but not in the two KO mice.

Our immunohistochemical data indicated that expression levels of both GluD2 and Cbln1 in *ts3* mutant cerebella were significantly reduced or undetectable, respectively, whereas the protein level of Car8 was comparable to that of WT controls (Fig. 9). However, the results were inconsistent with our western blotting data, showing that Cbln1 expression levels were comparable in *ts3* and control cerebella (Fig. S5). It had previously been reported that Cbln1 immunoreactivity was not detectable in the *grid2*-disrupted mice [36]. In contrast, expression levels of GluD2 protein are normal in Cbln1 mutant mice (our unpublished data). Taken together, our data raised the possibility that either *grid2* or a gene affecting *grid2* expression is mutated in *ts3* mutant mice, although the possibility that Cbln1 is mutated in *ts3* mice could not be excluded. We subsequently conducted further examination by sequencing and PCR, resulting in identification of the *grid2* deletion, ranging from exon 3 to 8. Discrepancy of phenotypes between *ts3* and other *grid2* mutants suggests that the *ts3* mutation, which disrupts the majority of the coding region but retains N-terminal portion of the protein, causes phenotypic differences when compared with other mutants. It has been reported that the N-terminal domain of GluD2 (Arg321-Trp339) [37], which is deleted in *ts3* mutants, is crucial for Cbln1 binding and induction of presynaptic differentiation, suggesting at least that GluD2^ts3^ does not function through binding to Cbln1. Alternatively, there is a possibility that another gene is also mutated in *ts3* mutants. Although our observations indicated that only homozygous *ts3* mice, but not heterozygotes or WTs, showed phenotypic abnormalities, this does not entirely exclude the possibility that mutations occur on multiple genes in a linkage group on the same chromosome. Regarding any inconsistency between our immunohistochemical and western blotting data on Cbln1 expression, one possible explanation is that Cbln1 protein was washed away during the process of immunostaining due to lack of normal PF–PC synapse formation, as suggested in a previous report [36].

In this study, we also found individual phenotypic differences between *ts3* mutants, although their genetic background is almost identical. First, whereas most of those we observed developed paraplegia, some mice developed mild appendicular ataxia, including fore limbs. Rates of ataxic progression also displayed individual differences. Second, severity of cerebellar atrophy also differed between mice, which does not significantly correlate with difference in behavioral abnormalities. Third, some mice showed anterior folial atrophy in which the anterior part of the cerebellar folia was significantly smaller in size than its posterior counterpart (Fig. 2B, 4A), although this does not seem to directly correlate with severity in ataxia (Fig. 4A). In other *ts3* mutants, however, neither the anterior part nor the whole cerebella were smaller than those of WTs.

In summary, the present study demonstrated that the *ts3* mutant is a SCD model that exhibits unique features in its behavioral and neuropathological abnormalities when compared with other known SCD model mice. Further studies, including an electrophysiological approach, might address the fundamental question of whether the mutated gene is involved in development, survival, or playing a role in a signaling pathway in the cerebellar neural network.

## Supporting Information

Figure S1 **Reduced bodyweights in** ***ts3*** **mutant mice.** The average bodyweight (BW) of *ts3* mutants and WT controls at 4, 8, and 16 weeks of age (n = 3, means +/−SD). ***P<0.001, *P<0.05. Note that the average BWs are significantly lower in *ts3* mutants than in normal controls at all ages examined.
(TIF)

Figure S2 **Relative cerebellar size is significantly smaller in** ***ts3*** **mutants.** Cerebral and cerebellar sizes were measured from MR images as shown in Fig. 2. **A)** MR images of the WT and *ts3* brains with long and short axes as indicated by red lines to quantify cerebral size. **B)** Average cerebral size was measured by multiplying length of long and short axes (mm, n = 3). **C)** To examine whether relative cerebellar size is smaller than that of the cerebrum, ratios of average cerebellar and cerebral sizes (cerebellum/cerebrum, n = 3) in the two different genotypes are shown. Note that relative cerebellar size (cerebellum/cerebrum) is significantly smaller in *ts3* mutant mice than that of WT controls, although entire brain size is also smaller. **D)** Average body weight for mice used in the experiments are shown (n = 3). *P<0.05, ***P<0.001.
(TIF)

Figure S3 **Spinal cord morphology at different levels along antero-posterior axis.** Coronal sections of *ts3* and WT spinal cord were H&E stained to confirm normal spinal cord morphology for both genotypes, as shown in Fig. 2.
(TIF)

Figure S4 **Significant reduction in the average number of Purkinje cells in older** ***ts3*** **mutants.** Average number of Purkinje cells in each lobule (number/mm^2^) in *ts3* mutant and control mice at 4 weeks (**A**) and 1 year old (**B**). In 1-year-old *ts3* mutant mice, significant decreases in PC cells were observed in lobules VII and VIII of the mutant cerebella when compared with WT controls. **P<0.01.
(TIF)

Figure S5 **Expression level of Cbln1 in** ***ts3*** **cerebellum is comparable with that of control.** Western blotting on *ts3/+* and *ts3/ts3* cerebella demonstrated that Cbln1 is expressed at comparable levels in *ts3* mutants and controls, whereas GluD2 is not detectable in *ts3* cerebella. This is in contrast to the results shown in Fig. 9 by immunohistochemistry, where Cbln1 was not detectable in *ts3* cerebella.
(TIF)

Figure S6 **Detection of a large deletion of the** ***grid2*** **DNA and GluD2 protein in** ***ts3*** **mutant mice.** Sequencing analysis revealed a 1001 bp deletion in the *ts3 grid2* cDNA corresponding to sequences from exon 3 to 8 (highlighted in **A**), causing deletion of 333 amino acids (83-415) in the GluD2 protein (highlighted in **B**). This DNA deletion also results in a frame-shift mutation, and the final gene product is 124 amino acids in length (see Fig. 10B).
(TIF)
