# Supplementary figures and images for: A New Mouse Allele of Glutamate Receptor Delta 2 with Cerebellar Atrophy and Progressive Ataxia
Source: PLoS One. 2014 Sep 24;9(9):e107867. doi: 10.1371/journal.pone.0107867 (PMC4176021; doi:10.1371/journal.pone.0107867)

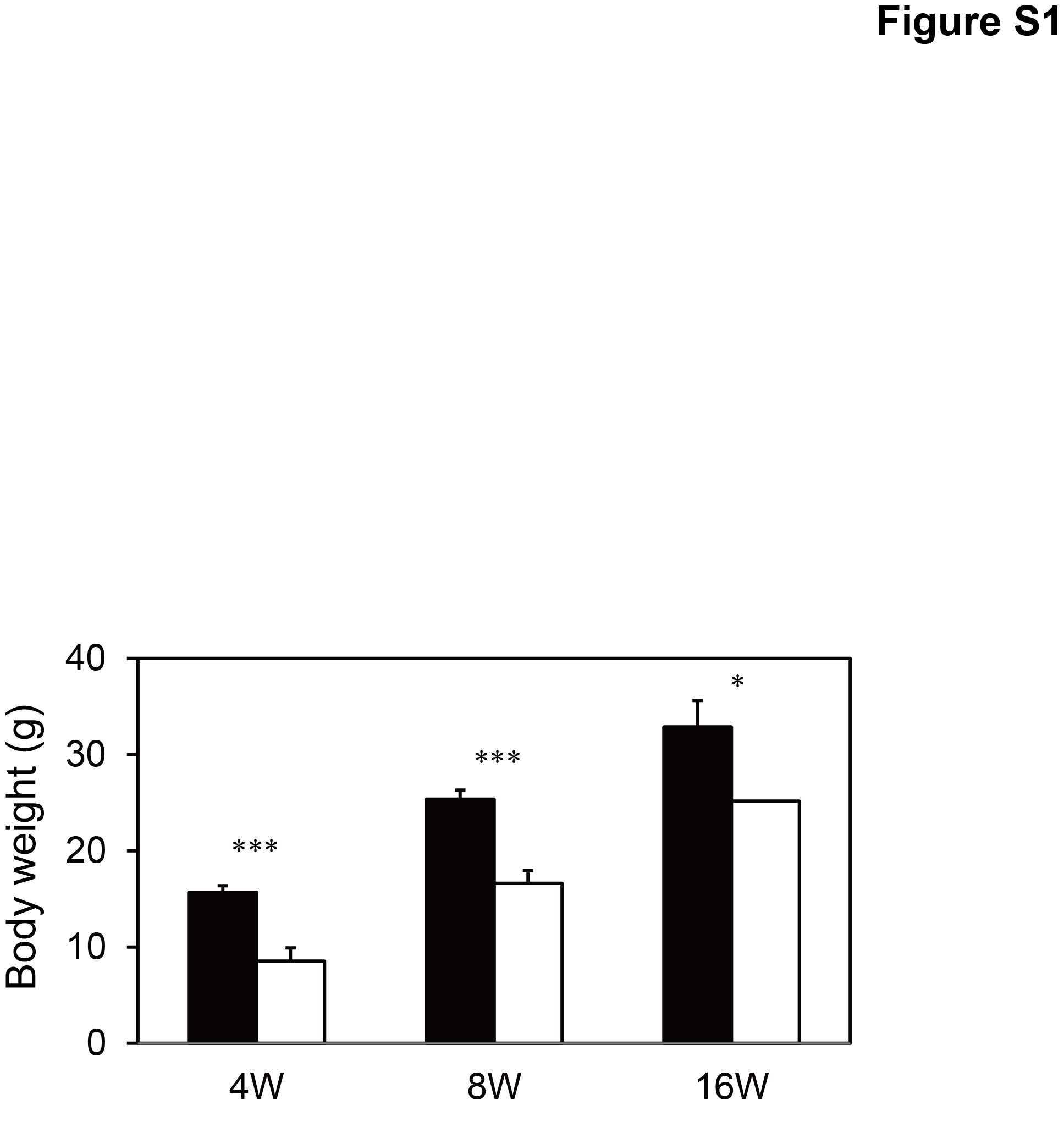

Supplement: Figure S1 — Reduced bodyweights in ts3 mutant mice. The average bodyweight (BW) of ts3 mutants and WT controls at 4, 8, and 16 weeks of age (n = 3, means +/−SD). ***P<0.001, *P<0.05. Note that the average BWs are significantly lower in ts3 mutants than in normal controls at all ages examined. (TIF) [file pone.0107867.s001.tif]

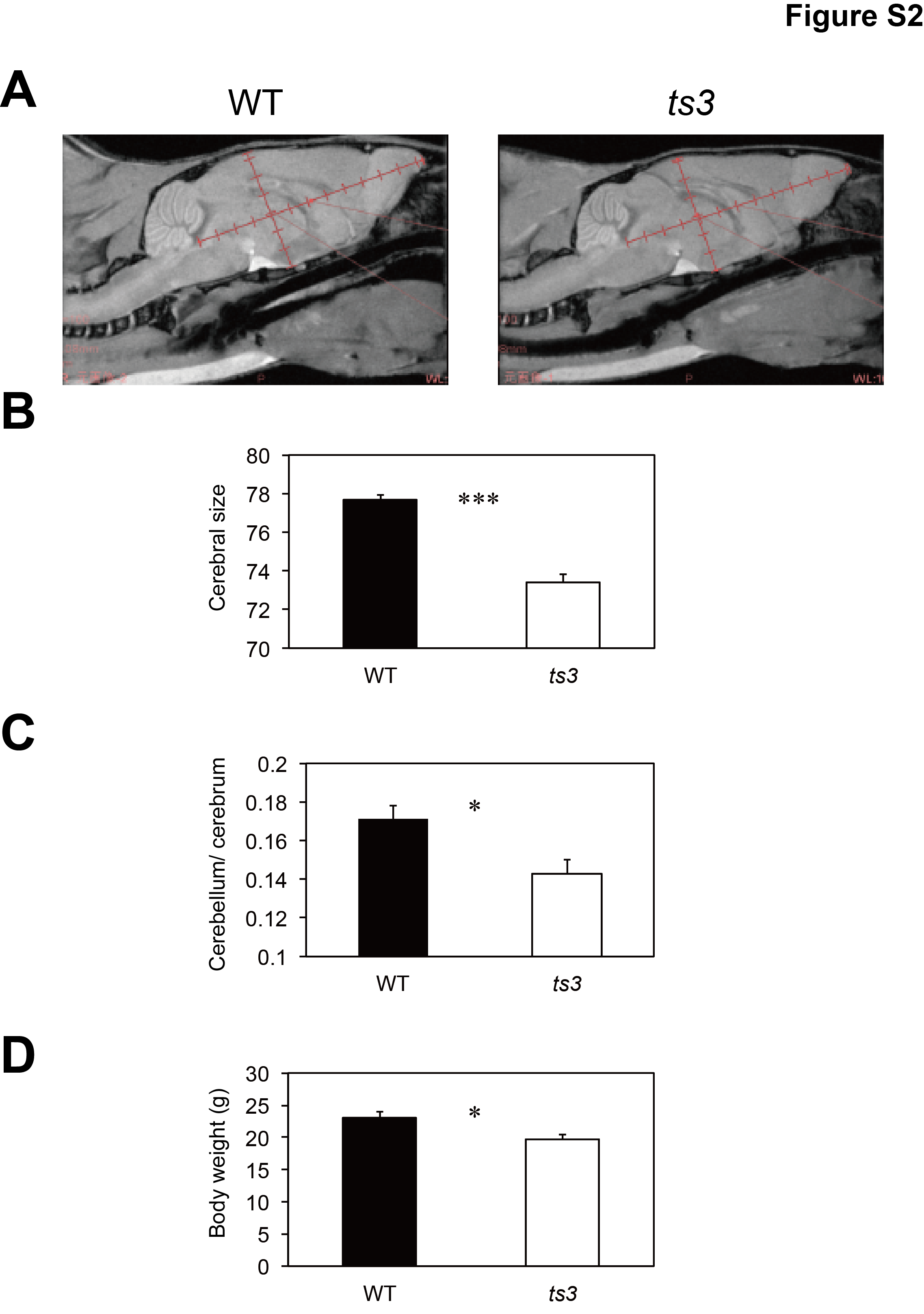

Supplement: Figure S2 — Relative cerebellar size is significantly smaller in ts3 mutants. Cerebral and cerebellar sizes were measured from MR images as shown in Fig. 2. A) MR images of the WT and ts3 brains with long and short axes as indicated by red lines to quantify cerebral size. B) Average cerebral size was measured by multiplying length of long and short axes (mm, n = 3). C) To examine whether relative cerebellar size is smaller than that of the cerebrum, ratios of average cerebellar and cerebral sizes (cerebellum/cerebrum, n = 3) in the two different genotypes are shown. Note that relative cerebellar size (cerebellum/cerebrum) is significantly smaller in ts3 mutant mice than that of WT controls, although entire brain size is also smaller. D) Average body weight for mice used in the experiments are shown (n = 3). *P<0.05, ***P<0.001. (TIF) [file pone.0107867.s002.tif]

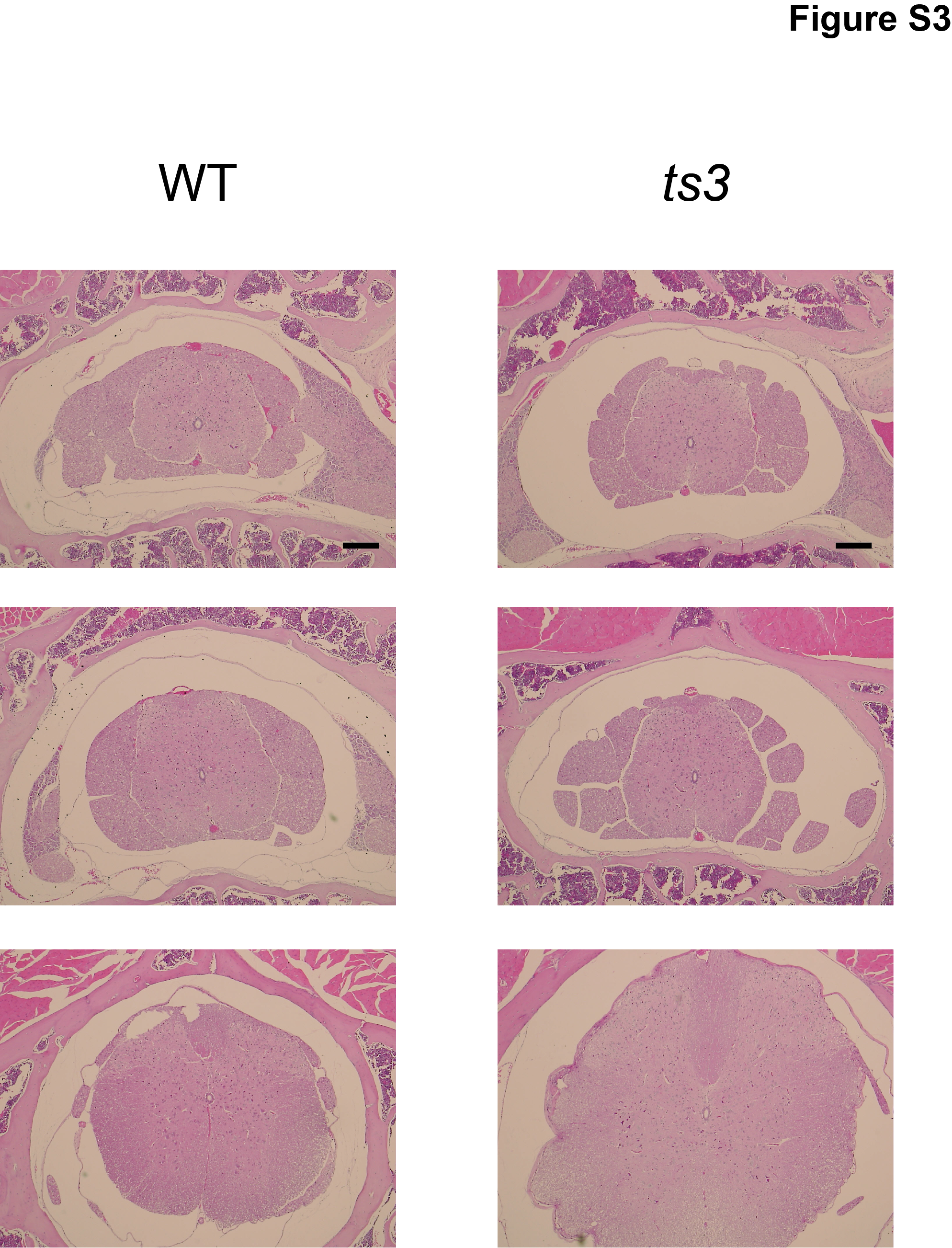

Supplement: Figure S3 — Spinal cord morphology at different levels along antero-posterior axis. Coronal sections of ts3 and WT spinal cord were H&E stained to confirm normal spinal cord morphology for both genotypes, as shown in Fig. 2. (TIF) [file pone.0107867.s003.tif]

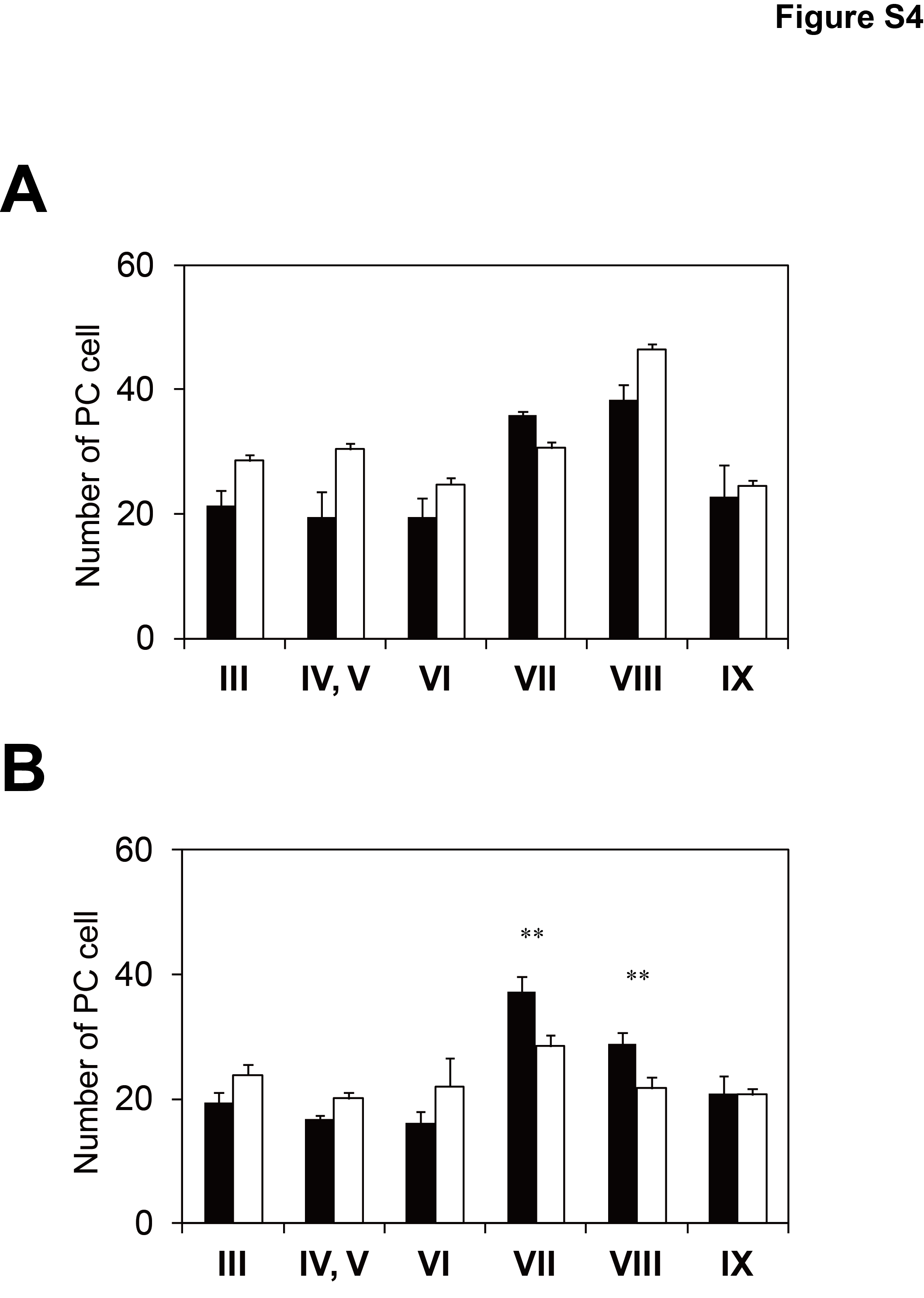

Supplement: Figure S4 — Significant reduction in the average number of Purkinje cells in older ts3 mutants. Average number of Purkinje cells in each lobule (number/mm2) in ts3 mutant and control mice at 4 weeks (A) and 1 year old (B). In 1-year-old ts3 mutant mice, significant decreases in PC cells were observed in lobules VII and VIII of the mutant cerebella when compared with WT controls. **P<0.01. (TIF) [file pone.0107867.s004.tif]

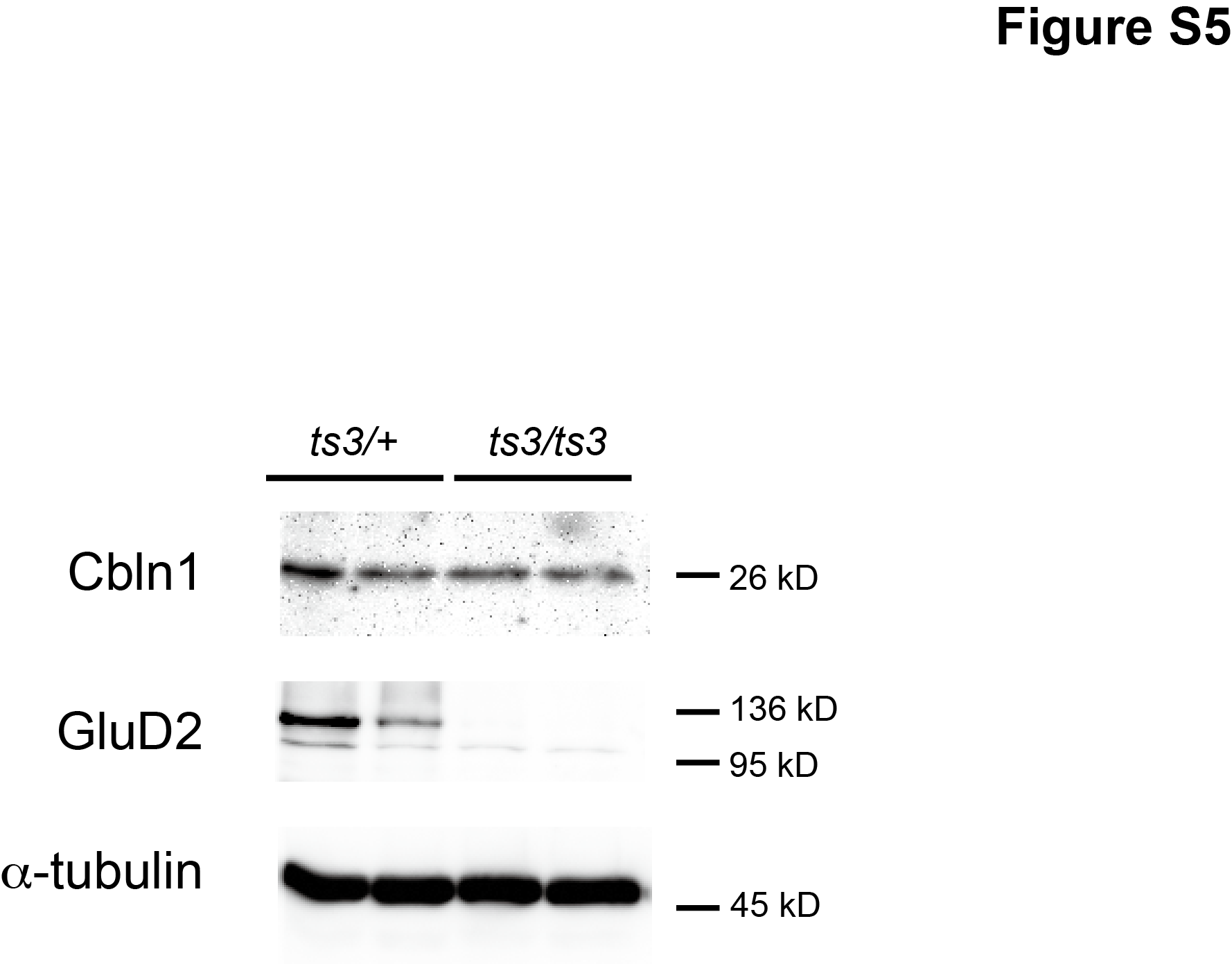

Supplement: Figure S5 — Expression level of Cbln1 in ts3 cerebellum is comparable with that of control. Western blotting on ts3/+ and ts3/ts3 cerebella demonstrated that Cbln1 is expressed at comparable levels in ts3 mutants and controls, whereas GluD2 is not detectable in ts3 cerebella. This is in contrast to the results shown in Fig. 9 by immunohistochemistry, where Cbln1 was not detectable in ts3 cerebella. (TIF) [file pone.0107867.s005.tif]

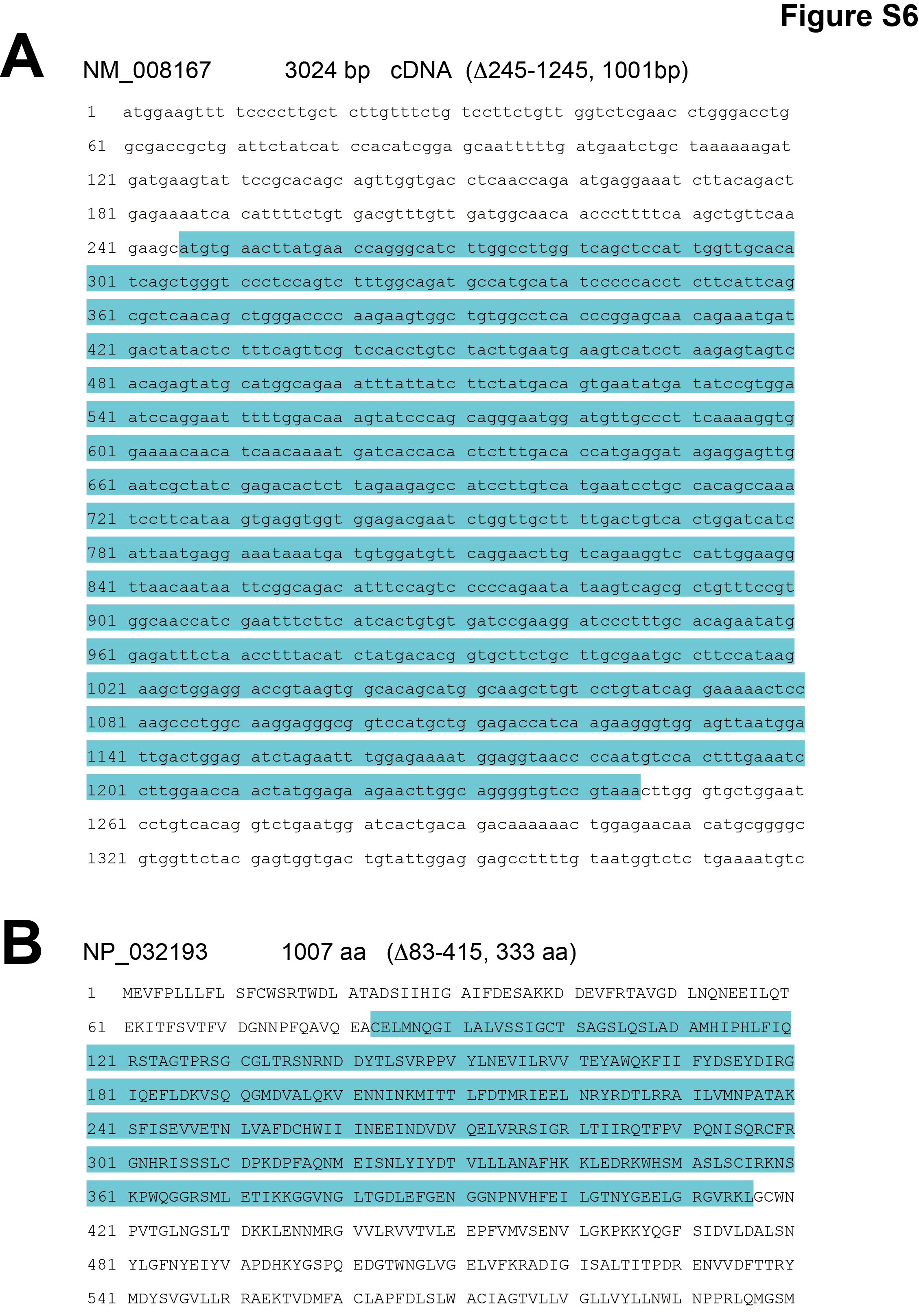

Supplement: Figure S6 — Detection of a large deletion of the grid2 DNA and GluD2 protein in ts3 mutant mice. Sequencing analysis revealed a 1001 bp deletion in the ts3 grid2 cDNA corresponding to sequences from exon 3 to 8 (highlighted in A), causing deletion of 333 amino acids (83-415) in the GluD2 protein (highlighted in B). This DNA deletion also results in a frame-shift mutation, and the final gene product is 124 amino acids in length (see Fig. 10B). (TIF) [file pone.0107867.s006.tif]
